# A Young Adult With Essential Thrombocythemia Presenting as Myocardial Infarction

**DOI:** 10.7759/cureus.28883

**Published:** 2022-09-07

**Authors:** Nagapratap Ganta, Ankita Prasad, Smriti Kochhar, Kajal Ghodasara, Sandeep Pavuluri, Arthur Okere, Pramil Cheriyath

**Affiliations:** 1 Internal Medicine, Hackensack Meridian Health Ocean University Medical Center, Brick, USA; 2 Medical School, Hackensack Meridian Health Ocean University Medical Center, Brick, USA; 3 Medical School, Rowan University School of Osteopathic Medicine, Stratford, USA; 4 Cardiology, Hackensack Meridian Health Ocean University Medical Center, Brick, USA

**Keywords:** atypical megakaryocytes, stemi, myocardial infarction, hemorrhage, thrombus, platelet, mutation, jak 2, essential thrombocyathemia

## Abstract

Essential thrombocythemia (ET) is a myeloproliferative neoplasm involving the clonal proliferation of platelets. It is Philadelphia negative and is associated with Janus kinase 2 (JAK2), calreticulin (CALR), or myeloproliferative leukemia virus oncogene (MPL) mutations. The resultant platelets have quantitative and qualitative defects, making them more sticky and prone to thromboembolism. However, ET does not only affect platelet survival, it also has a low leukemogenic potential. It's more common in the elderly, 60 years or more, but can be seen in all age groups, including children. Patients with ET have an increased risk of vascular events like hemorrhage and thromboses like cerebrovascular events, myocardial infarction, superficial thrombophlebitis, deep vein thrombosis, and pulmonary embolism. Cardiovascular risk factors like hypertension, diabetes, and smoking can lead to increased thromboembolism and atherosclerosis. The management of ET focuses primarily on the prevention of thrombosis and hemorrhage. It involves cardiovascular risk management and antiplatelet and cytoreductive therapy according to the risk stratification. Low-risk ET patients are treated with low-dose aspirin, and high-risk ET patients are treated with cytoreductive therapy with hydroxyurea. Interferon (IFN) and anagrelide are reserved for young patients or pregnant women. This case report discusses a 40-year-old male, a known smoker presenting with myocardial infarction and left anterior descending artery (LAD) blockage without any prior history. His high platelets and the relative absence of cardiovascular risk factors helped reach the diagnosis, and bone marrow analysis and mutation analysis confirmed the diagnosis. The patient was started on hydroxyurea, which decreased the total platelet count.

## Introduction

Essential thrombocythemia (ET) is a myeloproliferative neoplasm (MPN) with excessive clonal platelet production and megakaryocytic hyperplasia of the bone marrow. The incidence varies from 0.2-2.5 per 100,000 people per year, with a prevalence of 38-57 cases per 100,000 [1], as the survival is slightly worse than the general population. Janus kinase 2 (JAK2) V617F, an acquired gain-of-function mutation, is the most common mutation, present in 50-60% of patients [2]. Most cases have a mutation in JAK2, calreticulin (CALR), or myeloproliferative leukemia virus oncogene (MPL) [3]. Most cases of ET are sporadic, and familial cases are thought to be due to a predisposition to acquire somatic mutations rather than germline mutations. The World Health Organization defines essential thrombocytosis as having a platelet count greater than 450,000/mm3 and either a JAK2, CALR, or MPL mutation without clonal or reactive causes [4]. Its incidence increases with age, and the median age at diagnosis is 60 years, but up to 20 % of patients may be younger than 40. It is seen more in females, with an incidence ratio of 2:1 [5]. Patients with ET have an increased risk of vascular events like hemorrhage and thromboses like cerebrovascular events, myocardial infarction, superficial thrombophlebitis, deep vein thrombosis, and pulmonary embolism. It is most likely due to qualitative defects in platelets in addition to the increased numbers. However, platelet survival is not affected in ET. The management of ET focuses on the prevention of thrombosis and hemorrhage. It involves cardiovascular risk management and antiplatelet and cytoreductive therapy according to the risk stratification. 

## Case presentation

**Figure 1:**
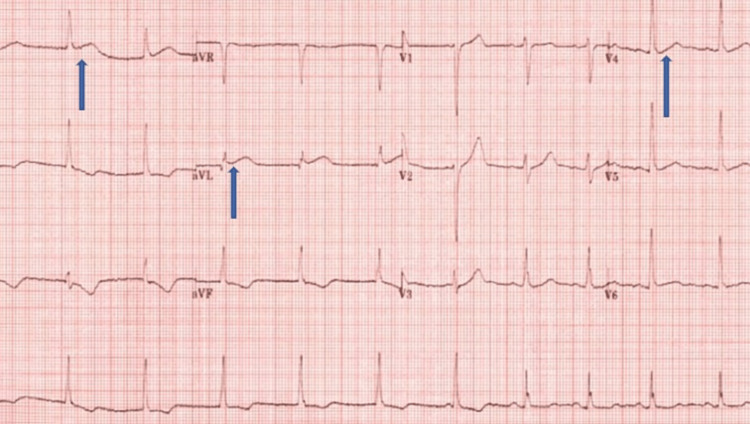
ECG at presentation showing ST elevation in leads I, aVL, and V3

**Figure 2:**
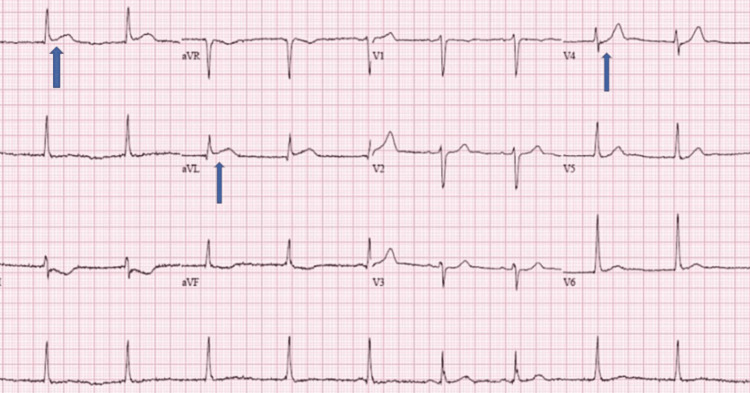
ECG showing ST elevation in I, aVl, and V3

Our patient is a 38-year-old male who presented with retrosternal chest pain after returning home from walking his dog. He described the chest pain as stabbing and being present in the mid-chest, non-radiating, and associated with paresthesia in the extremities, shortness of breath, diaphoresis, nausea, and vomiting. He took some antacid, which did not help, so he called 911. He had no similar episodes in the past. At presentation, he was alert, anxious, and sweaty with a heart rate of 90/minute, blood pressure was 145/88 mm Hg, respiration rate was 18/minute, and saturation of peripheral oxygen (SpO2) was 97% at room air. EKG in the emergency room (ER) showed ST-elevation myocardial inflammation (STEMI) with ST-segment elevations in the leads I, aVL, and V3 with reciprocal changes in the inferior leads (Figures 1, 2)

**Figure 3:**
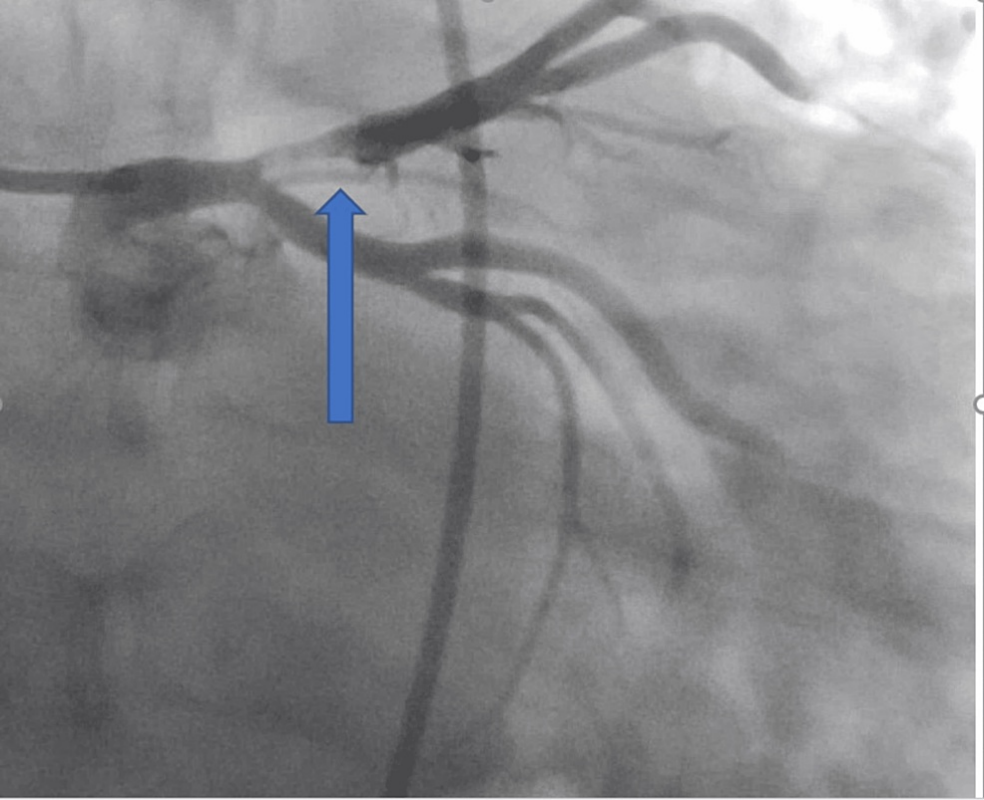
Angiography Images, showing LAD blockage (blue arrow) LAD: left anterior descending artery

**Figure 4:**
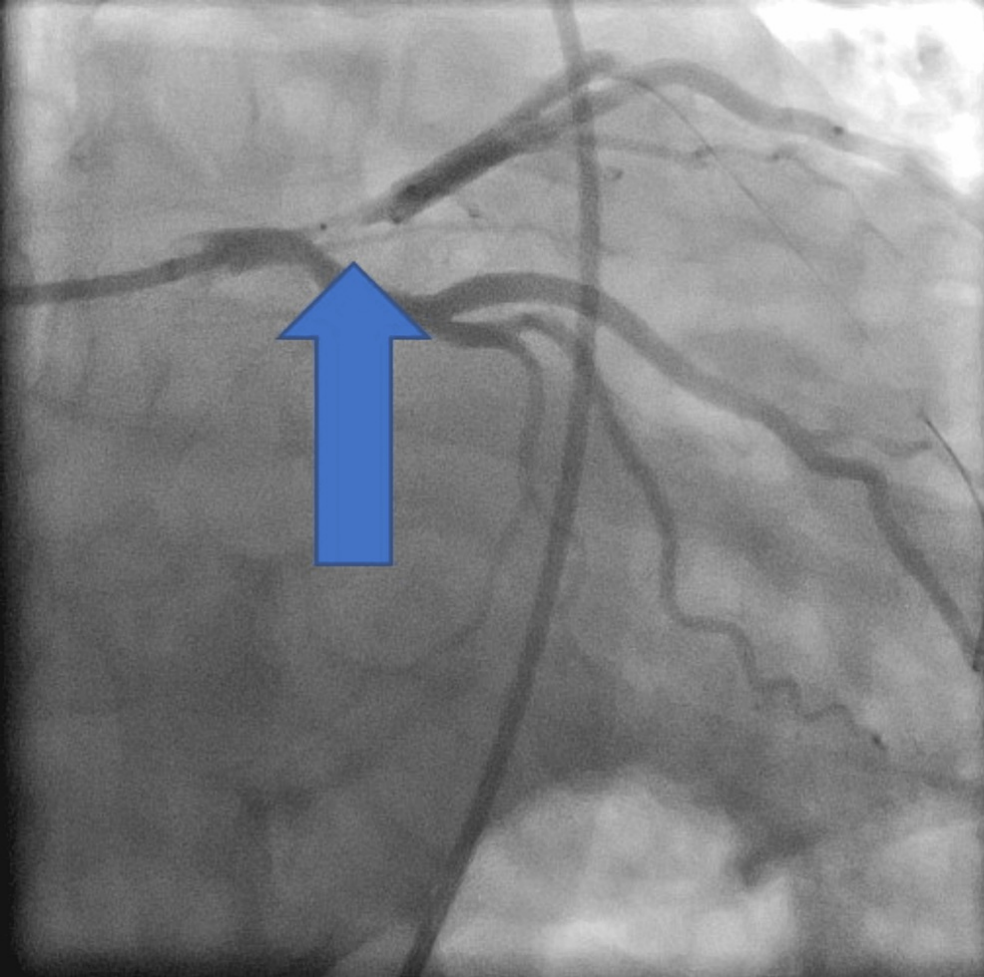
Angiography image LAD blockage (blue arrow) LAD: left anterior descending artery

He was taken to the catheterization lab for percutaneous coronary intervention (PCI), which demonstrated 100% flush occlusion of the left anterior descending artery (LAD) at the ostium of the vessel with collaterals already emanating from the right system; PCI also demonstrated minimal luminal disease (Figures 3, 4)

He had a stent put in the LAD following balloon dilatation. He did not have any significant past medical or surgical history. His personal history was marked by smoking and alcohol abuse (one pint of vodka per day). However, he was off alcohol for four months and was on naltrexone and venlafaxine for smoking and alcohol abuse. His family history was significant for his mother's hypertension and his father's hyperlipidemia.

**Figure 5:**
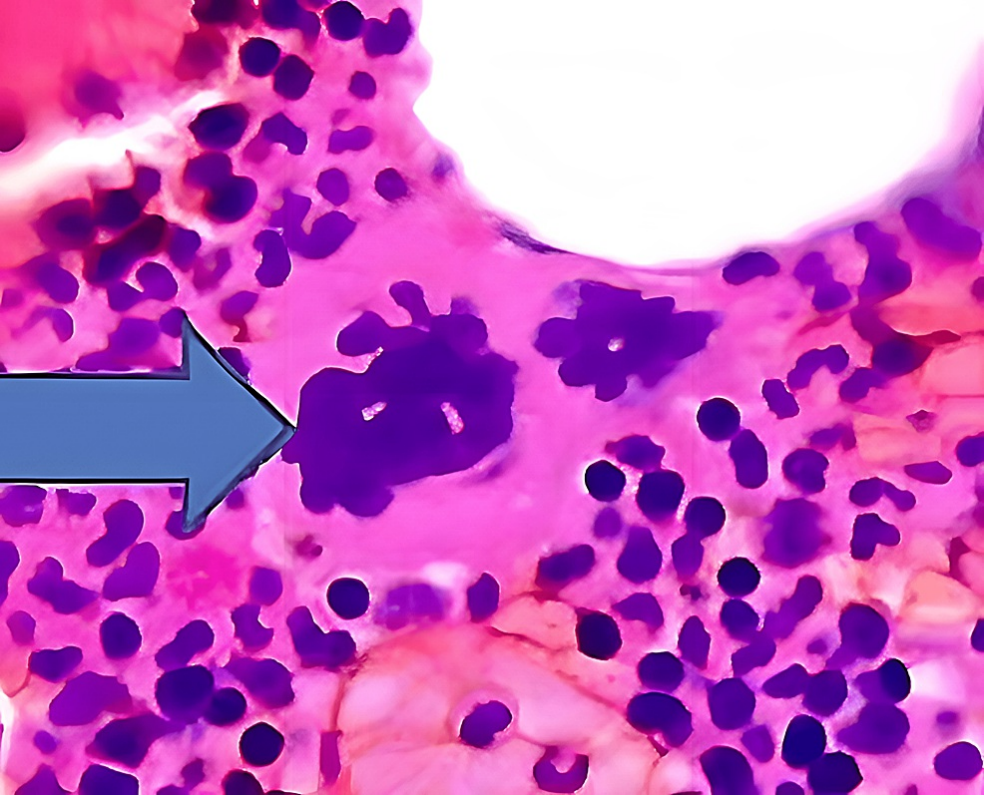
Histopathology slide showing cellular bone marrow with atypical hypolobulated megakaryocytes (blue arrow)

The complete blood count (CBC) was significant, with a red blood cell count of 5.72x10*6/ul, hemoglobin of 16.4g/dl, hematocrit of 48.2, and platelet count of 7x10*6/ul. There was no previous baseline CBC available to compare. The comprehensive metabolic panel was normal; troponin was elevated (0.25), international normalized ratio (INR) was 1.8, and prothrombin time 13.9 seconds. We investigated him for the high platelet count, and he was found to have the JAK2 V617F mutation. Bone marrow biopsy, aspirate smears, and flow cytometry showed a cellular bone marrow with maturing trilineage hematopoiesis and large atypical hypolobated megakaryocytes (Figure 5).

The morphology of the atypical megakaryocytes, along with the clinical history of the positive JAK2 mutation, was suggestive of ET. No other clinically significant mutations were detected. Based on these, cytoreductive and antiplatelet therapy were started with hydroxyurea, aspirin, and ticagrelor. He is now receiving atorvastatin, metoprolol for managing cardiovascular risk factors, smoking cessation support, and nitroglycerine as needed for chest pain. His most recent lab work after the start of the treatment was significant for ​​red blood cells 5.33 (4.50-5.30 10*6/uL), hemoglobin 15.4 (13.2-17.5 g/dL), and platelets 578 (140-450 10*3/uL).

## Discussion

Diagnosis of ET involves complete blood analysis with peripheral blood smear, cytogenetic studies for a JAK2 mutation, and if negative, CALR or MPL mutation analysis and bone marrow aspirate and biopsy. Besides these, other causes of secondary thrombocytosis and other myeloproliferative neoplasms must be excluded. The platelet count is > 450,000/ul (> 4.5× 10*9/ul) but can be >1,000,000/ul (> 1x107cells/ul). The platelet count may decrease during pregnancy [6]. The peripheral smear may show giant platelets and megakaryocyte fragments. The clinical features are due to vascular occlusions involving small vessels of the distal extremities (causing erythromelalgia), the eye (causing ocular migraine), or the central nervous system and coronary system. The incidence of acute coronary disease in patients with ET is 9.4% [7]. In a study with a series of 891 patients with World Health Organization-defined ET followed for a median of 6.2 years, the rate of fatal or nonfatal thrombotic events was 1.9 per 100 patient-years [8]. Among the significant thrombotic complications, studies have shown that arterial thrombus is more common than venous thrombus [8], and arterial thrombosis is responsible for the great majority of complications, including ischemic stroke, myocardial infarction, and peripheral arterial occlusion. There is a wide range of reported incidences of thrombosis (9-22%) and hemorrhage (3-37%) at the time of ET diagnosis [9,10]. After diagnosis, reported rates for subsequent thrombosis and hemorrhage, over a median follow-up duration of 3 to 11 years, are 7-31% and 8-14%, respectively [9,10].

Patients are at high risk of thrombosis if they are over the age of 60 or have a history of thrombosis, and they are at increased risk of bleeding if their platelet counts are more significant than 1500 x 10*9/L. ET diagnoses are based on bone marrow morphology and genetic tests for mutation. A study by Posfai et al. on ET-related vascular complications revealed JAK2 V617F mutation-positive status in most cases (10/14, 71.4%) of myocardial infarction and in all patients who suffered from other major arterial thrombotic complications, such as ET-related stroke. In addition, at least one or more vascular risk factors, such as smoking, hypertension, diabetes, and hyperlipidemia, were present in most patients with myocardial infarction complications [11]. Thus, maintaining strict control of cardiovascular risk factors like smoking, hypertension, and hyperlipidemia is important in ET patients to prevent thrombosis. The treatment of ET focuses on preventing thrombosis and bleeding while minimizing the risk of leukemogenic disease transformation. Low-risk ET patients are treated with low-dose aspirin, and high-risk ET patients are treated with cytoreductive therapy with hydroxyurea. Interferon (IFN) and anagrelide are reserved for young patients or pregnant women. Anagrelide is recommended as second-line therapy for resistant or intolerant to hydroxyurea patients. 

It is not uncommon for an ET patient to have coronary events and present with acute coronary syndrome (ACS), as in this patient. However, in ACS, cardiologists focus more on diagnosing and treating coronary artery disease, especially revascularization. The abnormality in platelet count can be overlooked, resulting in a missed diagnosis of ET in patients without notable thrombocytosis. The red flags for ET in myocardial infarction cases are high peripheral blood platelet count (> 450 X 10 9/L), even if only mildly above this level, no severe atherosclerotic narrowing found in coronary angiography, and the presence of thrombotic occlusion. Treatment involves cytoreduction, antithrombotic therapy, aspiration thrombectomy, and revascularization with distal protection to prevent distal embolization. Cytoreduction is advised before revascularization to prevent platelet activation and further episodes of thrombosis [11]. All these patients are classified as high-risk ET. A previous study showed that a history of thrombosis at diagnosis was significantly associated with recurrent thrombosis. In some cases, another thrombotic event occurred despite treatment with antiplatelet drugs and hydroxyurea [12]. 

## Conclusions

It is not uncommon for an ET patient to have coronary events and present with ACS, as in this patient. However, the urgent need for diagnosing and revascularization takes precedence leading to the missed diagnosis of ET if it is their first clinical presentation or the platelets are only marginally elevated. The presence of a thrombotic coronary artery with minimal or no preceding cardiovascular risk factors and an elevated platelet count should be considered potential red flags for ET. Timely diagnosis and management are essential as cytoreductive therapy is indicated before revascularization with distal protection to prevent distal embolization. In addition to antiplatelet and cytoreductive therapy in ET patients, strict control of cardiovascular risk factors is vital to prevent thrombosis. ET patients can have recurrent major thrombotic events even with optimal antiplatelet and antithrombotic therapies.
