# Correction: A Young Adult With Essential Thrombocythemia Presenting as Myocardial Infarction

**DOI:** 10.7759/cureus.c76

**Published:** 2022-10-05

**Authors:** Nagapratap Ganta, Ankita Prasad, Smriti Kochhar, Kajal Ghodasara, Sandeep Pavuluri, Arthur Okere, Pramil Cheriyath

**Affiliations:** 1 Internal Medicine, Hackensack Meridian Health Ocean University Medical Center, Brick, USA; 2 Medical School, Hackensack Meridian Health Ocean University Medical Center, Brick, USA; 3 Medical School, Rowan University School of Osteopathic Medicine, Stratford, USA; 4 Cardiology, Hackensack Meridian Health Ocean University Medical Center, Brick, USA

This article has been corrected to include Dr. Arthur Okere as sixth author and remove the acknowledgement of Dr. Okere. Despite having a very active role in caring for the patient and consulting with the submitting author regarding the case, Dr. Okere was omitted by the submitting author, Dr. Nagapratap Ganta. Dr. Okere was not made aware of this article until after it was published, at which point he contacted the journal with details regarding the above, which Dr. Ganta subsequently confirmed. As a result, Dr. Arthur Okere has been added as an author. Dr. Nagapratap Ganta has communicated his deep regret for this error in judgment and is in full agreement regarding the addition of Dr. Okere.

